# Serum Uric Acid Is a Mediator of the Association Between Obesity and Incident Nonalcoholic Fatty Liver Disease: A Prospective Cohort Study

**DOI:** 10.3389/fendo.2021.657856

**Published:** 2021-05-13

**Authors:** Qian Zhang, Xiaoqian Ma, Jie Xing, Haiyun Shi, Runkuan Yang, Yue Jiao, Shuohua Chen, Shouling Wu, Shutian Zhang, Xiujing Sun

**Affiliations:** ^1^Department of Gastroenterology, National Clinical Research Center for Digestive Disease, Beijing Friendship Hospital, Capital Medical University, Beijing Digestive Disease Center, Beijing Key Laboratory for Precancerous Lesion of Digestive Disease, Beijing, China; ^2^Clinical Epidemiology and EBM Unit, National Clinical Research Center for Digestive Disease, Beijing Friendship Hospital, Capital Medical University, Beijing, China; ^3^Department of Emergencies and Critical Care, Oslo University Hospital, Oslo, Norway; ^4^Department of Cardiology, Kailuan General Hospital, Tangshan, China

**Keywords:** serum uric acid, body mass index, mediation analysis, metabolic syndrome, nonalcoholic fatty liver disease, obesity

## Abstract

**Objective:**

Obesity has been demonstrated to show a consistent link with the increased possibility of nonalcoholic fatty liver disease (NAFLD). Since both serum uric acid (SUA) and obesity are essential components of metabolic syndrome (MetS), it is uncertain whether the incidence of NAFLD results from serum uric acid, obesity, or other potential factors based on previous studies.

**Patients and methods:**

This study enrolled 16,839 participants with no history of alcohol consumption and no fatty liver disease in 2010. All participants completed a survey which included health and lifestyle questionnaires, and underwent physical examination, ultrasonography, and laboratory examinations of blood samples. After the four-year follow up, 5,104 (30.31%) participants were diagnosed with NAFLD. The associations between SUA, BMI or obesity, and incident NAFLD were assessed by multivariate linear regression, logistic regression analysis, and mediation analysis, respectively.

**Results:**

By adjusting demographic and serum characteristics, linear correlation coefficients between obesity and SUA were 20.26 [95% confidence interval (CI)]: 15.74, 24.77), 13.31 (95% CI: 6.63, 19.99) and 22.21 (95% CI: 16.41, 28.02) in the total population, and in the female and male groups, respectively. The odds ratios were 2.49 (95% CI: 1.61, 3.87) in the total population, 5.71 (95% CI: 2.25, 14.45) in the female group and 1.99 (95% CI: 1.15, 3.45) in the male group for the correlation between obesity and incident NAFLD. The mediation analysis showed that SUA contributed to 10.03%, 0.58%, and 12.54% of obesity-related NAFLD development in the total population, females and males, respectively.

**Conclusion:**

The findings showed mediation linkages of both obesity and SUA with the incident NAFLD. The role of SUA as a mediator constitutes clinical significance that should be recognized and considered.

## Introduction

Nonalcoholic fatty liver disease (NAFLD) refers to the presence of hepatic steatosis without any other causes for secondary hepatic fat accumulation such as heavy alcohol consumption. NAFLD is now subdivided into nonalcoholic fatty liver (NAFL) and nonalcoholic steatohepatitis (NASH); the boundary is whether there is evidence of significant inflammation (1). Strong relationships between NAFLD and insulin resistance (IR), type 2 diabetes mellitus (T2DM), metabolic syndrome (MetS), cardiovascular disease, and other chronic diseases have been proven (2). Previous studies have showed that more than one third of diabetic patients have NAFLD (3, 4), and bidirectionally, NAFLD is associated with a two-fold risk of incident T2DM and MetS (5). Besides, NAFLD conferred an OR of 1.64 ([95% confidence interval (CI)]: 1.26, 2.13) for cardiovascular disease (6), which showed that NAFLD is also associated with an increased risk of cardiovascular disease (7).

Serum uric acid (SUA) levels are the result of purine levels as well as alterations due to lipid metabolism and glucose (8, 9). Several pieces of evidence have shown that SUA levels are correlated with NAFLD onset and progression (10). At the same time, serum levels of uric acid and the prevalence of NAFLD dramatically increase with measures of obesity. Although the incidence of NAFLD and high uric acid is related to body mass index (BMI), it is still unknown whether uric acid affects the incidence of BMI-related NAFLD through some potential pathways. Previous study has also suggested a significant role of elevated SUA in the pathogenesis of NAFLD (8). There is also no evidence available on the possible pathways through which obesity, SUA, and NAFLD interact. From a hemodynamic perspective, reductions in weight and BMI may have a significant impact on SUA, and renal urate excretion suggests that changes in weight may play a role in the regulation of SUA levels. SUA levels may increase blood pressure, triglyceride, and insulin levels while also decreasing cholesterol levels, which could lead to the onset of NAFLD (10). In this context, researchers have hypothesized that SUA mediates the positive association between obesity and NAFLD. Consistent with this hypothesis, a recent review illustrated the mechanisms linking obesity, SUA, and NAFLD based on clinical studies (11). However, this hypothesis has not been tested in large-scale prospective cohort studies. Elucidating the associations among obesity, SUA, and NAFLD is significant because obesity-related hyperuricemia leads to increased societal and economic burdens of obesity-associated NAFLD and mortality. Thus, we used multivariate analyses to confirm the connections among obesity, SUA, and NAFLD. Furthermore, mediation methods were used to examine the connections and associations among SUA, obesity, and NAFLD within the context of a large community-based cohort that included 16,839 adults.

## Material and Methods

### Patient Enrollment and Setting

The Kailuan Longitudinal Study is an ongoing epidemiologic prospective cohort (trial registration number: ChiCTR-TNC-11001489, registration site: http://www.chictr.org.cn/index.aspx) (12–14) of all employees (≥18 years of age, including those who retired) who received the Kailuan Group’s detailed and thorough medical examination at Tangshan City, China. The employees underwent questionnaire investigations and clinical and laboratory examinations every two years. The data collected from the Kailuan Longitudinal Study started in 2010.

For this study, 92,967 participants completed the three consecutive surveys between 2010 and 2014. All the participants received questionnaire surveys, clinical and laboratory examinations in 2010, 2012, and 2014, respectively. During the four-year period, the demographic and clinical characteristics showed limited changes, within 15%, in both participants with obesity and without obesity (**Supplementary Figure 1**). The results showed the smoking rates were 30-31% in the obesity group and 26-27% in the non-obesity group over four years, which also showed stable trends.

Briefly, the present study excluded participants who fulfilled the following exclusion criteria: (i) abnormal or missing ultrasound examination results with fatty liver disease at baseline in 2010; (ii) missing liver ultrasound examination results in the follow-up; (iii) history of chronic hepatitis C or HBsAg positivity, alcohol intake, long-term medication intake, or clinical medical diagnostic history of liver cirrhosis; and (iv) missing laboratory data or demographic survey data. The completed survey included health and lifestyle questionnaires, physical examination, ultrasonography, and laboratory examinations of blood samples. A standardized self-administered questionnaire surveyed the medical history and lifestyle factors of all participants. In cases in which some participants had difficulties in completing the questionnaire, a nurse provided assistance.

The study was approved by the Ethics Committees of Kailuan Hospital (ethical approval number: 2006-5) in compliance with the Declaration of Helsinki. All participants signed written informed consent.

### Clinical Variables Collection

Participants underwent their clinical examination in the morning after an overnight fast. The examination included blood pressure assessment, anthropometry, and physical examination by a physician. Demographic data regarding gender, age, smoking and alcohol use, physical activity, and medical history (e.g., diabetes and hypertension) were collected using a self-reported questionnaire (12, 14–16). There were also clinical and detailed measurements of height and weight with a stadiometer and balance beam scale. Additionally, BMI was calculated by dividing weight in kilos by height squared in meters (kg/m^2^) (17). Obesity was defined as BMI ≥ 28 kg/m^2^ (18). Three measurements of systolic blood pressure (SBP) were then assessed at five-minute intervals after a five-minute rest using a standardized sphygmometer in the seated position; we used the average of three measurements.

### Lab Testing

All participants had no food intake for more than eight hours before the physical examination. Elbow venous blood was taken in the early morning to determine the levels of SUA, serum creatinine (Cr), triglyceride (TG), fasting blood glucose (FBG), total cholesterol (TC), and C-reactive protein (CRP). Venous blood samples were also collected after an overnight eight-hour timeframe, followed by transfer of the samples into EDTA-containing vacuum tubes. The endpoint test method was used to measure total TC levels, while the glycerol phosphate oxidase method was used for TG level measurements. For FBG levels, the hexokinase/glucose-6-phosphate dehydrogenase method was used for the measurements. Moreover, an autoanalyzer (Hitachi 747; Hitachi, Tokyo, Japan) at the central laboratory of Kailuan General Hospital analyzed the samples (15, 19, 20).

### Outcome Definition

The outcome was determined by analyzing images obtained from abdominal realtime ultrasonography (Philips HD-15, Bothell, WA) with a transducer frequency of 2.50–3.50 MHz by trained technicians; these images provided the basis for the measurement of fatty liver disease. After an overnight fast, the participants were examined in a supine position or a left lateral position with the right upper quadrant of the abdomen exposed. An image server was used to store all the ultrasonographic images. Two doctors examined the liver morphology, size, envelope, boundary, internal echo spot, distribution, liver contour display, and intrahepatic tubular structure and finally recorded the results of the examination.

According to the ultrasonographic diagnostic criteria of NAFLD of the Study Group of Liver and Metabolism, Chinese Society of Endocrinology, signs or evidence of at least two of the following factors served as the basis of the diagnosis of fatty liver: (1) hypoechogenicity in the far field of the liver; (2) hyperechogenicity in the near field of the liver or bright liver, as well as signs of it being stronger than the kidney cortex; and (3) a blurry intrahepatic tubular structure (9). The diagnostic criteria for NAFLD were as follows: (a) a lack of evidence such as alcohol consumption and long-term use of medication and (b) ultrasound findings of fatty liver disease. Alcohol intake in this study was defined as any form of alcohol intake (e.g., beer, white spirit with a high or low content of aromatics, and wine) (21).

### Statistical Methods

Continuous variables, such as age and BMI, were described as the mean ± standard deviation (SD), followed by a comparison using Student’s t test. Other continuous variables were described as medians (interquartile ranges, IQRs) and compared by the Mann-Whitney U-test. Categorical variables, such as gender and education, were described as percentages and were compared using the χ^2^ test. Multivariate linear regression analysis was used to assess the association between SUA, BMI, and obesity. The covariates of age, gender, work type, education level, physical exercise, serum Cr, TG, FBG, cholesterol and CRP were adjusted. The β value and 95% CI were estimated. Multivariate logistic regression analysis was further used to assess the association between the incidence of NAFLD and BMI or obesity, respectively. Covariates such as age, gender, work type, education level, physical exercise, serum Cr, TG, FBG, cholesterol and CRP were included in this analysis. The odds ratio (OR) and 95% confidence interval (CI) were estimated.

Given the associations between SUA, obesity, and incident NAFLD, a mediation analysis was conducted to examine how SUA as a mediator impacted the association between obesity (the independent variable) and incident NAFLD (the outcome variable). The total effect (the association between BMI or obesity and NAFLD), direct effect (the total effect without the influence of SUA), and indirect effect (the effect of BMI or obesity on the outcome of NAFLD attributable to SUA) were assessed with adjusted variables of age, gender, work type, education level, physical exercise, serum Cr, TG, FBG, cholesterol, and CRP. Percent mediation was calculated by dividing the indirect effect by the total effect and represented the proportion of the total effect attributable to the mediator (22). The statistical analyses and tests adopted a two-tailed P value of 0.05 or less as an indication of statistical significance. All statistical analyses were performed with SAS 9.4 (SAS Institute Inc., Cary, NC, USA).

## Results

### The Baseline Information and Outcomes of NAFLD

**Figure 1:**
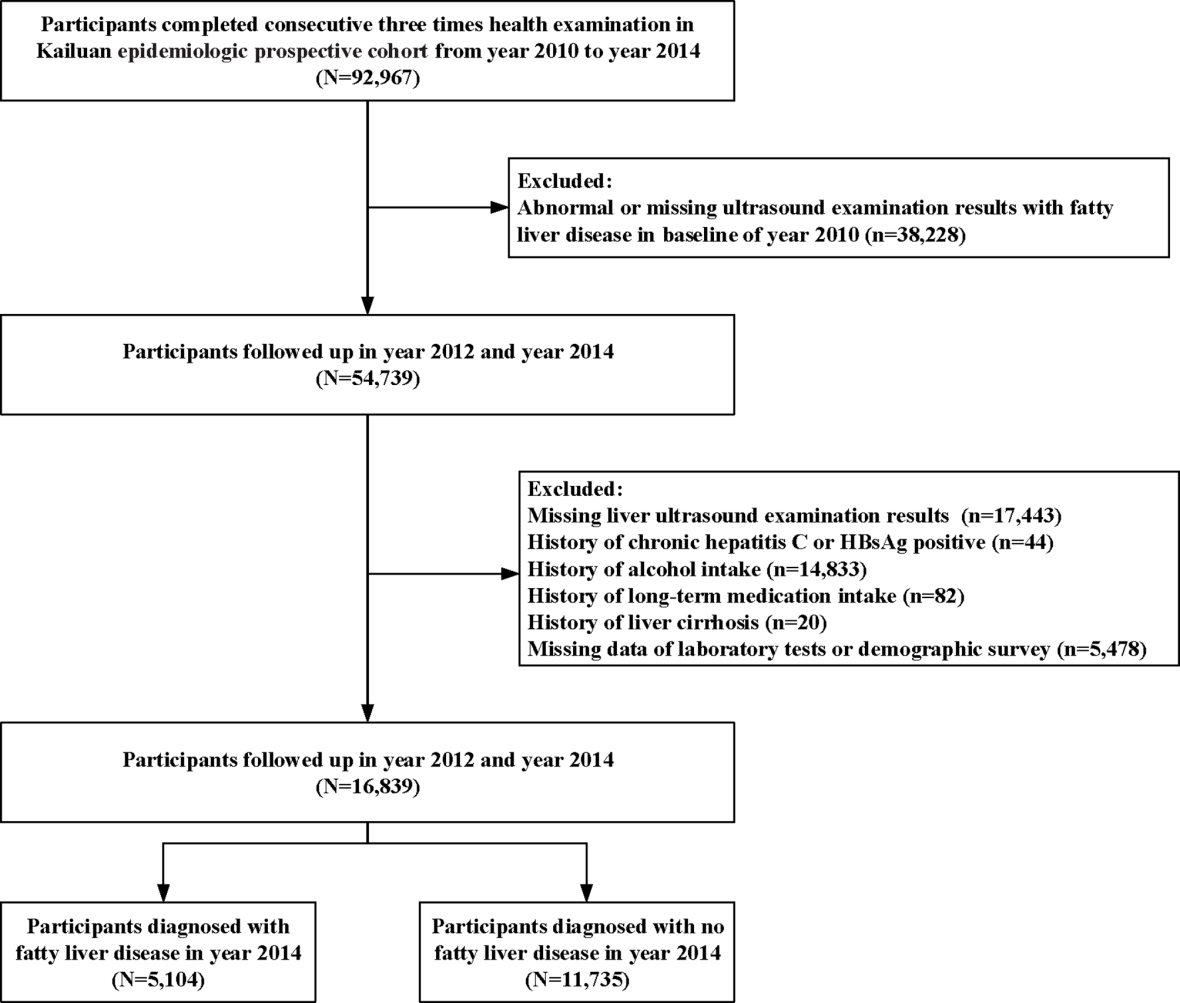
The flow chart of the enrolled participants.

**Tables 1** and **2** show the baseline information of the participants in the study divided by gender and NAFLD. A total of 16,839 individuals (5,418 females and 11,421 males) who met the inclusion criteria were included in the analysis (**Figure 1**). After four years of follow-up, 5,104 participants were diagnosed with nonalcoholic fatty liver disease. The male group showed a higher percentage of incident NAFLD than the female group (31.41% *vs*. 28.00%) after four years (P<0.01).

**Table 1:** Demographic and serum biomarker information of the participants with no NAFLD in baseline in the Kailuan cohort study.

Demographic Characteristics		Total (n=16,839)	Female (n=5,418)	Male (n=11,421)	P-value
Age, years, mean ± SD		50.62 ± 12.95	48.57 ± 11.82	51.60 ± 13.34	<0.01
BMI, kg/m^2^, mean ± SD		23.79 ± 2.94	23.43 ± 3.07	23.97 ± 2.86	<0.01
Obesity, BMI≥28kg/m^2^		1,277 (7.58)	382 (7.05)	895 (7.84)	0.07
Non-obesity, BMI<28kg/m^2^		15,562 (92.42)	5,036 (92.95)	10,526 (92.16)	
Smoking, n (%)	Never or ever	12,203 (72.47)	5,365 (99.02)	6,838 (59.87)	<0.01
	Current	4,636 (27.53)	53 (0.98)	4,583 (40.13)	
Marital Status, n (%)	Married	16,196 (96.18)	5,224 (96.42)	10,972 (96.07)	0.27
	Divorced, single or widowed	643 (3.82)	194 (3.58)	449 (3.93)	
Work Type, n (%)	Mental	2,705 (16.06)	1,490 (27.50)	1,215 (10.64)	<0.01
	Physical	14,134 (83.94)	3,928 (72.50)	10,206 (89.36)	
Education Level, n (%)	Below high school	11,567 (68.69)	3,232 (59.65)	8,335 (72.98)	<0.01
	High school or above	5,272 (31.31)	2,186 (40.35)	3,086 (27.02)	
Physical Activity, n (%)	No	5,129 (30.46)	1,538 (28.39)	3,591 (31.44)	<0.01
	Yes	11,710 (69.54)	3,880 (71.61)	7,830 (68.56)	
SUA, µmol/L, median (IQR)		263.00 (214.00, 318.00)	228.00 (193.10, 272.00)	281.00 (229.00, 338.00)	<0.01
SBP, mmHg, mean ± SD		125.96 ± 18.83	120.63 ± 19.13	128.49 ± 18.14	<0.01
DBP, mmHg, mean ± SD		81.62 ± 10.35	78.24 ± 10.24	83.23 ± 10.01	<0.01
TG, mmol/L, median (IQR)		1.11 (0.82, 1.51)	1.05 (0.76, 1.41)	1.15 (0.85, 1.56)	<0.01
TC, mmol/L, median (IQR)		4.72 (4.17, 5.28)	4.70 (4.15, 5.38)	4.73 (4.18, 5.23)	0.56
FBG, mmol/L, median (IQR)		5.18 (4.80, 5.62)	5.03 (4.69, 5.48)	5.20 (4.86, 5.70)	<0.01
CRP, mg/L, median (IQR)		0.90 (0.40, 2.01)	0.83 (0.40, 1.83)	0.90 (0.31, 2.14)	0.46
Cr, µmol/L, median (IQR)		78.50 (66.00, 94.52)	66.00 (56.00, 79.00)	84.00 (72.60, 99.10)	<0.01
Incident NAFLD, n (%)	No	11,735 (69.69)	3,901 (72.00)	7,834 (68.59)	<0.01
	Yes	5,104 (30.31)	1,517 (28.00)	3,587 (31.41)	

BMI, Body Mass Index; Cr, Creatinine; CRP, C-reactive Protein; FBG, Fasting Blood Glucose; IQR, interquartile range; NAFLD, Nonalcoholic Fatty Liver Disease; SBP, Systolic Blood Pressure; SD, Standard Deviation; SUA, Serum Uric Acid; TC, Total Cholesterol; TG, Triglyceride.

**Table 2:** Baseline demographic and serum biomarker information of the NAFLD patients and normal participants in the Kailuan cohort study.

Demographic Characteristics		Incident NAFLD (n=5,104)	Without Incident NAFLD (n=11,735)	P-value
Age, years, mean ± SD		51.04 ± 12.53	50.44 ± 13.12	0.01
BMI, kg/m^2^, mean ± SD		25.09 ± 2.83	23.23 ± 2.81	<0.01
Obesity, BMI≥28kg/m^2^		716 (14.03)	561 (4.78)	<0.01
Non-obesity, BMI<28kg/m^2^		4,388 (85.97)	11,174 (95.22)	
Gender, n (%)	Female	1,517 (29.72)	3,901 (33.24)	<0.01
	Male	3,587 (70.28)	7,834 (66.76)	
Smoking, n (%)	Never or ever	3,553 (69.61)	8,650 (73.71)	<0.01
	Current	1,551 (30.39)	3,085 (26.29)	
Marital Status, n (%)	Married	4,900 (96.00)	11,296 (96.26)	0.43
	Divorced, single or widowed	204 (4.00)	439 (3.74)	
Work Type, n (%)	Mental	681 (13.34)	2,024 (17.25)	<0.01
	Physical	4,423 (86.66)	9,711 (82.75)	
Education Level, n (%)	Below high school	3,719 (72.86)	7,848 (66.88)	<0.01
	High school or above	1,385 (27.14)	3,887 (33.12)	
Physical Activity, n (%)	No	1,511 (29.60)	3,618 (30.83)	0.11
	Yes	3,593 (70.40)	8,117 (69.17)	
SUA, µmol/L, median (IQR)		275.00 (219.00, 330.00)	258.00 (212.00, 312.30)	<0.01
SBP, mmHg, mean ± SD		128.70 ± 18.38	124.77 ± 18.90	<0.01
DBP, mmHg, mean ± SD		83.32 ± 10.21	80.89 ± 10.33	<0.01
TG, mmol/L, median (IQR)		1.24 (0.90, 1.80)	1.08 (0.78, 1.41)	<0.01
TC, mmol/L, median (IQR)		4.83 (4.26, 5.70)	4.69 (4.12, 5.21)	<0.01
FBG, mmol/L, median (IQR)		5.20 (4.84, 5.70)	5.14 (4.78, 5.60)	<0.01
CRP, mg/L, median (IQR)		1.17 (0.50, 2.70)	0.80 (0.30, 1.77)	<0.01
Cr, µmol/L, median (IQR)		80.00 (68.00, 94.50)	78.00 (65.00, 94.60)	<0.01

BMI, Body Mass Index; Cr, Creatinine; CRP, C-reactive Protein; FBG, Fasting Blood Glucose; IQR, interquartile range; NAFLD, Nonalcoholic Fatty Liver Disease; SBP, Systolic Blood Pressure; SD, Standard Deviation; SUA, Serum Uric Acid; TC, Total Cholesterol; TG, Triglyceride.

Compared with females, the included males were three years older (51.60 ± 13.34 years *vs*. 48.57 ± 11.82 years, P<0.01). In males, the BMI was 23.97 ± 2.86 kg/m^2^, versus 23.43 ± 3.07 kg/m^2^ in females (P<0.01). The smoking rate (40.13% *vs*. 0.98%) and physical work rate (89.36% *vs*. 72.50%) were higher in males than in females (P<0.01). Conversely, the physical activity rate among females was 71.61%, which was higher than that among males (68.56%, P<0.01).

In addition to the differences in demographic information, males were almost significantly higher than females in all serum biomarkers, including SUA, SBP, TC, FBG, and Cr (P<0.01). The serum levels of TC and CRP were shown to be insignificant (P=0.56 and P=0.46). Due to the different values of demographic and serum information, the following analyses were all divided by gender to determine whether there were different trends in the incidence of NAFLD.

As shown in **Table 2**, almost all the baseline demographic and serum biomarkers showed significant differences between participants with incident NAFLD and those without incident NAFLD (all P<0.05), in addition to marital status (P=0.43) and physical activity (P=0.11). Participants with incident NAFLD after the four-year follow-up showed a higher BMI (25.09 ± 2.83kg/m^2^ *vs*. 23.23 ± 2.81kg/m^2^) and higher SUA levels (275 µmol/L *vs*. 258 µmol/L) than participants without NAFLD (P<0.01). Other serum biomarkers, such as TG, TC, and FBG of participants with incident NAFLD showed higher levels than those of participants without NAFLD (all P<0.01).

### Multivariate Linear Regression Between Obesity and SUA in Males and Females

Attempts were made to show the relationship between obesity (BMI ≥ 28 kg/m^2^) and SUA across different genders. As shown in the multivariate linear regression analyses in **Table 3**, by adjusting for age, gender, smoking, marital status, work type, education level, physical activity, TG, TC, FBG, SBP, CRP and Cr, the correlation coefficient between obesity and SUA was 20.26 (95% CI: 15.74, 24.77) in the total population. The correlation of obesity and SUA was higher in the male group (22.21, 95% CI: 16.41-28.02) than in the female group (13.31, 95% CI: 6.63-19.99). The **Supplementary Table 1** shows the results of linear regression between BMI and SUA in males and females.

**Table 3:** Linear regression of the influencing factors for SUA divided by gender.

Total population	β (95%CI)	P-value	Female	β (95%CI)	P-value	Male	β (95%CI)	P-value
Obesity	20.26 (15.74, 24.77)	<0.01		13.31 (6.63, 19.99)	<0.01		22.21 (16.41, 28.02)	<0.01
Age	-0.23 (-0.35, -0.12)	<0.01		0.30 (0.12, 0.47)	<0.01		-0.40 (-0.54, -0.25)	<0.01
Gender	57.61 (54.57, 60.64)	<0.01		–	–		–	–
Smoking	6.28 (3.32, 9.24)	<0.01		4.76 (-12.41, 21.92)	0.59		4.97 (1.73, 8.21)	<0.01
Marital status	34.03 (27.59, 40.47)	<0.01		23.43 (14.19, 32.67)	<0.01		38.18 (29.77, 46.58)	<0.01
Working type	-11.02 (-14.70, -7.34)	<0.01		-6.74 (-11.13, -2.36)	<0.01		-16.29 (-21.78, -10.79)	<0.01
Education level	0.32 (-2.85, 3.48)	0.84		5.95 (1.69, 10.22)	0.01		-3.52 (-7.79, 0.741)	0.11
Physical activity	-7.71 (-10.34, -5.09)	<0.01		-6.85 (-10.67, -3.03)	<0.01		-7.85 (-11.25, -4.45)	<0.01
TG	6.51 (5.53, 7.48)	<0.01		3.26 (2.12, 4.39)	<0.01		9.25 (7.79, 10.72)	<0.01
TC	2.87 (1.92, 3.82)	<0.01		2.17 (1.07, 3.26)	<0.01		2.95 (1.51, 4.40)	<0.01
FBG	-2.18 (-2.95, -1.42)	<0.01		-0.47 (-1.42, 0.48)	0.34		-3.62 (-4.70, -2.54)	<0.01
SBP	0.32 (0.24, 0.39)	<0.01		0.18 (0.07, 0.28)	<0.01		0.36 (0.27, 0.45)	<0.01
CRP	1.75 (1.50, 2.00)	<0.01		1.30 (0.90, 1.71)	<0.01		1.94 (1.64, 2.25)	<0.01
Cr	-0.31 (-0.36, -0.26)	<0.01		-0.13 (-0.22, -0.04)	<0.01		-0.37 (-0.44, -0.31)	<0.01

CI, Confidence Interval; Cr, Creatinine; CRP, C-reactive Protein; FBG, Fasting Blood Glucose; Obesity: body mass index≥28kg/m^2^; SBP, Systolic Blood Pressure; SUA, Serum Uric Acid; TC, Total Cholesterol; TG, Triglyceride.

### Multivariate Logistic Regression Between Obesity, SUA and NAFLDin Males and Females

**Table 4** shows the relationship between obesity, SUA, and incident NAFLD according to gender-specific groups. Both obesity and SUA showed a significant correlation with incident NAFLD. The ORs for obesity were 2.49 (95% CI: 1.61, 3.87) in the total population, 5.71 (95% CI: 2.25, 14.45) in the female group and 1.99 (95% CI: 1.15, 3.45) in the male group for the correlation between obesity and incident NAFLD. The ORs for SUA were all significant (P<0.01) in the total population, the females and the males, respectively. The **Supplementary Table 2** shows the results of logistic regression between BMI, SUA and NAFLD in males and females.

**Table 4:** Logistic regression for the association of obesity, SUA and NAFLD divided by gender.

Total population	OR (95%CI)	P-value	Female	OR (95%CI)	P-value	Male	OR (95%CI)	P-value
Obesity	2.49 (1.61, 3.87)	<0.01		5.71 (2.25, 14.45)	<0.01		1.99 (1.15, 3.45)	0.01
SUA	1.00 (1.00, 1.00)	<0.01		1.00 (1.00, 1.00)	<0.01		1.00 (1.00, 1.00)	<0.01
Age	1.00 (0.99, 1.00)	0.01		1.02 (1.01, 1.02)	<0.01		0.99 (0.99, 0.99)	<0.01
Gender	0.79 (0.72, 0.86)	<0.01		–	–		–	–
Smoking	1.15 (1.06, 1.25)	<0.01		1.22 (0.68, 2.20)	0.51		1.12 (1.03, 1.22)	0.01
Marital status	1.10 (0.91, 1.32)	0.32		1.18 (0.82, 1.69)	0.38		0.97 (0.78, 1.20)	0.75
Working type	1.15 (1.03, 1.28)	0.01		1.20 (1.01, 1.42)	0.04		0.98 (0.85, 1.13)	0.75
Education level	0.82 (0.75, 0.90)	<0.01		0.65 (0.55, 0.76)	<0.01		0.90 (0.80, 1.01)	0.06
Physical activity	1.11 (1.03, 1.20)	0.01		1.06 (0.92,1.22)	0.44		1.13 (1.03, 1.23)	0.01
TG	1.24 (1.20, 1.28)	<0.01		1.34 (1.24,1.45)	<0.01		1.19 (1.14, 1.24)	<0.01
TC	1.03 (1.00, 1.06)	0.03		1.01 (0.98,1.05)	0.48		1.03 (0.99, 1.07)	0.11
FBG	1.02 (1.00, 1.05)	0.03		1.04 (1.01,1.08)	0.03		1.01 (0.98, 1.04)	0.47
SBP	1.01 (1.01, 1.01)	<0.01		1.01 (1.00,1.01)	0.01		1.01 (1.00, 1.01)	<0.01
CRP	1.01 (1.01, 1.02)	<0.01		1.02 (1.01,1.04)	<0.01		1.01 (1.00, 1.02)	0.01
Cr	1.00 (1.00, 1.00)	<0.01		1.00 (1.00,1.01)	0.05		1.00 (1.00, 1.00)	0.03

CI, Confidence Interval; Cr, Creatinine; CRP, C-reactive Protein; FBG, Fasting Blood Glucose; Obesity: body mass index≥28kg/m^2^; OR, Odds Ratio; SBP, Systolic Blood Pressure; SUA, Serum Uric Acid; TC, Total Cholesterol; TG, Triglyceride.

### SUA Mediated Obesity-Related NAFLD

**Figure 2:**
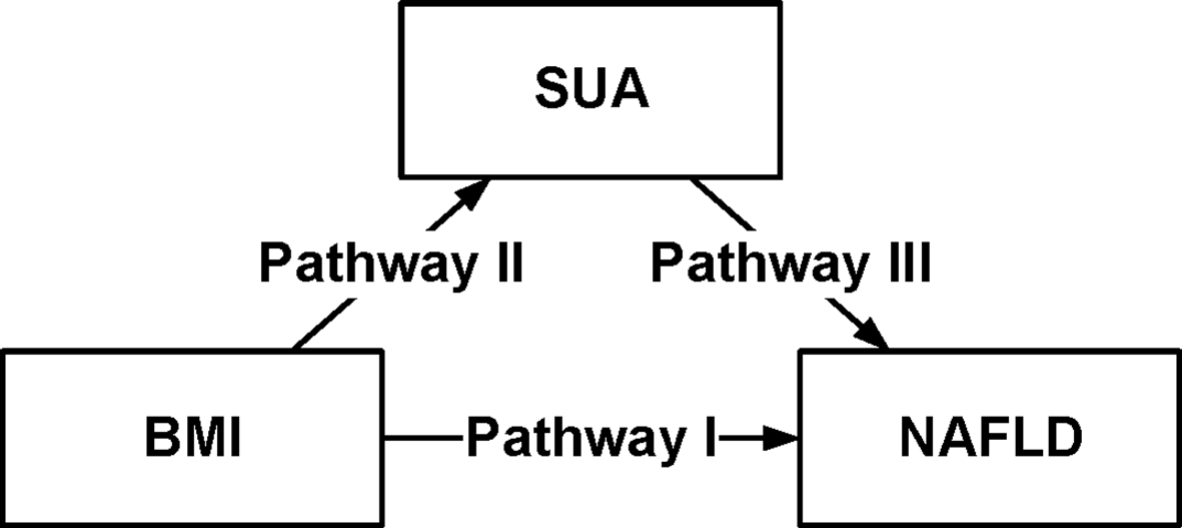
Conceptual version of the model used in this analysis. Serum Uric Acid (SUA) mediates the association between body mass index (BMI) and non-alcoholic fatty liver disease (NAFLD), in 16,839 individuals in the Kailuan Group. Pathway I represented the direct effect, pathway II and pathway III represented the indirect effect.

To characterize the effects of obesity on NAFLD that could be explained by a concomitant increase in serum uric acid, mediation analysis was performed to better understand the extent of the interactions. **Table 5** was obtained after performing and drawing a relationship triangle through the mediation analysis (**Figure 2**). The indirect effect is the effect that obesity has on nonalcoholic fatty liver through the mediator SUA. The direct effect is the amount of effect directly exerted on nonalcoholic fatty liver, irrespective of the mediator. Pathway I in **Figure 2** represents the direct effect, while pathways II and III together represent the indirect effect. Mediation analysis showed that SUA contributed to 10.03% of obesity-related NAFLD development. In the male group older than 45 years old, SUA accounted for 10.12% of the risk for obesity-related NAFLD development with significance (P<0.01). In contrast, in the female group, although SUA accounted for 6.79% of obesity-related NAFLD, the mediation effects seemed to be insignificant (P=0.19). In males, SUA mediated in different age groups. The mediation effect of SUA was significant in males, whether the age was older than 45 (15.28%) or less than 45 years old (10.12%) (P=0.12 and P<0.01). In females, the mediation effects were not significant both in older group (more than 45 years, P=0.19) and younger group (younger than 45 years, P=0.57).

**Table 5:** Mediation effect by SUA in the association between obesity and NAFLD.

	Indirect effect (Pathway II and III)	Direct effect (Pathway I)	Total effect	Mediation Percent (%)	P-value
Total (n=16,839)	1.07 (1.04, 1.10)	2.71 (2.40, 3.05)	2.90 (2.56, 3.28)	10.03	<0.01
Female (n=5,418)	1.00 (0.96, 1.05)	2.68 (2.14, 3.35)	2.68 (2.15, 3.35)	0.58	0.87
< 45 years (n=2,025)	0.97 (0.89, 1.07)	2.19 (1.29, 3.72)	2.14 (1.26, 3.61)	-4.93	0.57
>= 45 years (n=3,393)	1.05 (0.98, 1.12)	2.75 (2.13, 3.54	2.87 (2.21, 3.73)	6.79	0.19
Male (n=11,421)	1.09 (1.05, 1.14)	2.71 (2.35, 3.13)	2.95 (2.54, 3.44)	12.54	<0.01
< 45 years (n=3,238)	1.12 (1.02, 1.23)	2.90 (2.21, 3.81)	3.24 (2.45, 4.30)	15.28	0.02
>= 45 years (n=8,183)	1.07 (1.02, 1.12)	2.62 (2.21, 3.11)	2.80 (2.34, 3.36)	10.12	<0.01

All the mediation analyses were adjusted by age, gender, obesity, smoking status, marital status, work type, education level, physical activity, serum uric acid, systolic blood pressure, triglyceride, total cholesterol, fasting blood glucose, C-reactive protein and creatinine. Indirect effect: the effect of obesity on the outcome of non-alcoholic fatty liver disease attributable to the serum uric acid. Direct effect: the total effect without the influence of the serum uric acid. Total effect: the association between obesity and non-alcoholic fatty liver disease. Mediation Percent: the proportion of the total effect attributable to the serum uric acid.

## Discussion

Nonalcoholic fatty liver disease has become increasingly prevalent in recent years in Asia (23). We enrolled 16,839 participants who were free from NAFLD at the baseline examination in 2010. After the four-year follow-up, 5,104 (30.31%) participants were diagnosed with NAFLD. Our results showed that the obesity-related increased threat of NAFLD seemed to be affected by obesity-related changes related to SUA.

In many studies, the incidence of NAFLD increased with obesity, which is consistent with the notion that NAFLD is an obesity-related disorder (24). Consistent with our findings, previous studies have reported that SUA increases with increasing BMI (25) and, intriguingly, is also a risk factor for NAFLD (26). In a study including 166 compensated patients with a histological diagnosis of NAFLD, among which most patients were overweight or obesity, hyperuricemia was found to be associated with histological features of liver disease (27). In addition, a retrospective study of 118 consecutive biopsy-proven NAFLD patients showed that SUA was an independent predictor of NASH and its individual histological lesions, notably including fibrosis (28). Additionally, a meta-analysis of 25 studies found an approximately two-fold increased risk of NAFLD among subjects with hyperuricemia compared with subjects without hyperuricemia (29).

Another study by Zhang et al. showed that both uric acid levels and obesity contributed to the risk of NAFLD (30). In our four-year cohort study, the results coincided with Zhang’s findings. Both obesity (OR=2.49, P<0.01) and SUA (OR=1.00, P<0.01) showed significant correlation with incident NAFLD. The present study further used mediation analysis to evaluate the interactions between obesity, SUA and incident NAFLD in this longitudinal cohort with a large sample size. Mediation analysis showed that SUA contributed to 10% of obesity-related NAFLD development. This is consistent with the notion that increased SUA could be one of the main pathophysiological mechanisms for the increased prevalence of NAFLD in adults with obesity. Biological evidence has also suggested a role for IR, with its links to NAFLD that also showcase the role that IR plays in the accumulation of lipids in the liver (31). Equally important, there have been findings that both IR and hyperuricemia show commonality in certain directional causal impacts. IR induces hyperinsulinemia that could lead to the rise in reabsorption of renal urate through the stimulation of urate-anion exchange; thus, there is a reduction in the excretion of uric acid from the renal tubular cells due to insulin, increasing uric acid impacts pro-inflammatory action on adipocytes as well as stimulates cell proliferation, and the impairments in sensitivity to insulin and effectiveness of glucose of cells because of the impact to glucose indicate transport that is integral in obesity, and MetS (32, 33). From all of these findings, the examination of the links between SUA levels and NAFLD, as well as factors such as levels of insulin and obesity, entails a homeostasis model assessment involving resistance to insulin and levels of glucose that requires investigation and evaluation to expose the mechanisms, dynamics, and processes underlying the SUA and NAFLD relationship (34).

This is a large community-based prospective cohort study with a diagnosis of NAFLD performed by liver ultrasound, which is much more sensitive and specific than biomarkers such as fatty liver index. This study showed the association between incident NAFLD and SUA, which also indicated that obesity and hyperuricemia were linked to NAFLD incidence through mediation analysis. In these multivariate analyses, the confounding factors of obesity and NAFLD necessitate a level of controlled expectation to identify potential manipulable mechanisms. This is an important point because, first, obesity among people with hyperuricemia makes them more prone to increases in uric acid and body weight, leading to the need for these individuals to handle or manage both aspects. Second, another point of emphasis is the way that obesity and hyperuricemia influence the onset of NAFLD because of pathogenic processes. However, the mediation effect is significant in males, but not in females. The main reasons might be as follows: first, the prevalence of NAFLD in men is two times higher than that in women due to the protective effect of estrogen (11). On the other hand, men are more prone to abdominal obesity, which is directly related to the occurrence of NAFLD. Therefore, it can be concluded that SUA contributed to 10-15% of obesity-related NAFLD development in males, and that SUA acts as a mediator of both pathophysiology and clinical connection. When this theory is tested and affirmed, there will be the possibility to manage or treat NAFLD through restoration of the disrupted internal mechanisms within the body. This hypothesis has indicated that obesity-related hyperuricemia is reversible or can be treated with certain pharmacological and lifestyle interventions.

The study has some limitations. This current study was limited to Chinese demographics, specifically, those living in North China. Thus, the results of this study lack generalization to an extent due to different factors, but there is still the uniting or homogeneous aspect of our cohort that could help in reducing the lack of generalization to a more global context. The proportion of females was smaller than the males (32%; n=5,418); however, the large sample size still enabled us to conduct gender-specific analyses. Finally, though the participants enrolled from the epidemiologic prospective cohort, data on aspartate aminotransferase to calculate the scores on either NAFLD fibrosis score or fibrosis-4 score was not available. Nevertheless, participants with the clinical history of liver cirrhosis were excluded by searching for the clinical medical record.

## Conclusion

This large community-based longitudinal cohort study, in which potential confounding factors were adjusted for, provides a unique opportunity to understand the relationships of SUA with obesity-related NAFLD. The results suggested that patients living with obesity should pay attention to their uric acid levels to prevent NAFLD.

## Data Availability Statement

The original contributions presented in the study are included in the article/**Supplementary Material**. Further inquiries can be directed to the corresponding author.

## Ethics Statement

The study was approved by the ethics committees of Kailuan General Hospital (the ethical approval number: 2006-5), in compliance with the Declaration of Helsinki. The patients/participants provided their written informed consent to participate in this study.

## Author Contributions

SW and SZ conceived and designed the experiments. SC collected the data. SZ, XS, HS, YJ, and RY conducted the experiments. QZ, JX, and XM analyzed the data. QZ and XM drafted the manuscript. All authors contributed to the article and approved the submitted version.

## Funding

The study was supported by the Beijing Natural Science Foundation of China (7204249) and Beijing Talents Fund (2018000021469G198).

## Conflict of Interest

The authors declare that the research was conducted in the absence of any commercial or financial relationships that could be construed as a potential conflict of interest.

## Supplementary Material

Supplementary Table 1 Linear regression of the influencing factors for SUA divided by gender. BMI, Body Mass Index; CI, Confidence Interval; Cr, Creatinine; CRP, C-reactive Protein; FBG, Fasting Blood Glucose; SBP, Systolic Blood Pressure; SUA, Serum Uric Acid; TC, Total Cholesterol; TG, Triglyceride.

Supplementary Table 2 Logistic regression for the association of BMI, SUA and NAFLD divided by gender. BMI, Body Mass Index; CI, Confidence Interval; Cr, Creatinine; CRP, C-reactive Protein; FBG, Fasting Blood Glucose; OR, Odds Ratio; SBP, Systolic Blood Pressure; SUA, Serum Uric Acid; TC, Total Cholesterol; TG, Triglyceride.

## Abbreviations

BMI, Body Mass Index; CI, Confidence Interval; Cr, Creatinine; CRP, C-reactive Protein; FBG, Fasting Blood Glucose; IR, Insulin Resistance; MetS, Metabolic Syndrome; NASH, Nonalcoholic Steatohepatitis; NAFL, Nonalcoholic Fatty Liver; NAFLD, Nonalcoholic Fatty Liver Disease; OR, Odds Ratio; SBP, Systolic Blood Pressure; SD, Standard Deviation; SUA, Serum Uric Acid; T2DM, Type 2 Diabetes Mellitus; TC, Total Cholesterol; TG, Triglyceride.
