# Supplementary material for: Serum Uric Acid Is a Mediator of the Association Between Obesity and Incident Nonalcoholic Fatty Liver Disease: A Prospective Cohort Study
Source: Front Endocrinol (Lausanne). 2021 May 13;12:657856. doi: 10.3389/fendo.2021.657856 (PMC8158156; doi:10.3389/fendo.2021.657856)
Supplement: Supplementary Table 1 — Linear regression of the influencing factors for SUA divided by gender. BMI, Body Mass Index; CI, Confidence Interval; Cr, Creatinine; CRP, C-reactive Protein; FBG, Fasting Blood Glucose; SBP, Systolic Blood Pressure; SUA, Serum Uric Acid; TC, Total Cholesterol; TG, Triglyceride. [file Table_1.docx]

**Supplementary Table 1. Linear regression of the influencing factors for SUA divided by gender.**

| **Total population** | **β (95%CI)** | **P-value** | **Female** | **β (95%CI)** | **P-value** | **Male** | **β (95%CI)** | **P-value** |
| --- | --- | --- | --- | --- | --- | --- | --- | --- |
| BMI | 3.17 (2.75, 3.58) | <0.01 |  | 2.43 (1.85, 3.02) | <0.01 |  | 3.42 (2.86, 3.97) | <0.01 |
| Age | -0.24 (-0.36, -0.13) | <0.01 |  | 0.25 (0.08, 0.43) | <0.01 |  | -0.39 (-0.54, -0.25) | <0.01 |
| Gender | 56.89 (53.86, 59.91) | <0.01 |  | - | - |  | - | - |
| Smoking | 6.61 (3.67, 9.56) | <0.01 |  | 5.12 (-11.95, 22.20) | 0.56 |  | 5.44 (2.22, 8.67) | <0.01 |
| Marital status | 36.15 (29.74, 42.57) | <0.01 |  | 24.91 (15.71, 34.12) | <0.01 |  | 40.87 (32.49, 49.24) | <0.01 |
| Working type | -11.20 (-14.87, -7.53) | <0.01 |  | -6.78 (-11.15, -2.42) | <0.01 |  | -16.12 (-21.59, -10.64) | <0.01 |
| Education level | 1.10 (-2.05, 4.26) | 0.49 |  | 7.17 (2.92, 11.43) | <0.01 |  | -3.10 (-7.35, 1.15) | 0.15 |
| Physical activity | -8.00 (-10.62, -5.39) | <0.01 |  | -7.04 (-10.84, -3.23) | <0.01 |  | -8.16 (-11.55, -4.77) | <0.01 |
| TG | 5.87 (4.90, 6.85) | <0.01 |  | 2.91 (1.78, 4.05) | <0.01 |  | 8.45 (6.98, 9.92) | <0.01 |
| TC | 2.76 (1.82, 3.71) | <0.01 |  | 2.13 (1.04, 3.23) | <0.01 |  | 2.85 (1.41, 4.29) | <0.01 |
| FBG | -2.23 (-2.99, -1.46) | <0.01 |  | -0.44 (-1.39, 0.51) | 0.36 |  | -3.72 (-4.80, -2.64) | <0.01 |
| SBP | 0.24 (0.17, 0.32) | <0.01 |  | 0.12 (0.01, 0.22) | 0.03 |  | 0.29 (0.19, 0.38) | <0.01 |
| CRP | 1.73 (1.48, 1.97) | <0.01 |  | 1.23 (0.83, 1.64) | <0.01 |  | 1.94 (1.64, 2.25) | <0.01 |
| Cr | -0.32 (-0.37, -0.27) | <0.01 |  | -0.14 (-0.23, -0.05) | <0.01 |  | -0.38 (-0.44, -0.32) | <0.01 |

**Notes:**

**Abbreviations:** BMI, Body Mass Index; CI: Confidence Interval; Cr, Creatinine; CRP, C-reactive Protein; FBG, Fasting Blood Glucose; SBP, Systolic Blood Pressure; SUA, Serum Uric Acid; TC, Total Cholesterol; TG, Triglyceride.
