# Supplementary material for: Serum Uric Acid Is a Mediator of the Association Between Obesity and Incident Nonalcoholic Fatty Liver Disease: A Prospective Cohort Study
Source: Front Endocrinol (Lausanne). 2021 May 13;12:657856. doi: 10.3389/fendo.2021.657856 (PMC8158156; doi:10.3389/fendo.2021.657856)
Supplement: Supplementary Table 2 — Logistic regression for the association of BMI, SUA and NAFLD divided by gender. BMI, Body Mass Index; CI, Confidence Interval; Cr, Creatinine; CRP, C-reactive Protein; FBG, Fasting Blood Glucose; OR, Odds Ratio; SBP, Systolic Blood Pressure; SUA, Serum Uric Acid; TC, Total Cholesterol; TG, Triglyceride. [file Table_2.docx]

**Supplementary Table 2. Logistic regression for the association of BMI, SUA and NAFLD divided by gender.**

| **Total population** | **OR (95%CI)** | **P-value** | **Female** | **OR (95%CI)** | **P-value** | **Male** | **OR (95%CI)** | **P-value** |
| --- | --- | --- | --- | --- | --- | --- | --- | --- |
| BMI | 1.31 (1.25, 1.38) | <0.01 |  | 1.35 (1.23, 1.48) | <0.01 |  | 1.29 (1.22,1.37) | <0.01 |
| SUA | 1.01 (1.00, 1.01) | <0.01 |  | 1.01 (1.00, 1.02) | 0.01 |  | 1.01 (1.00,1.01) | <0.01 |
| Age | 1.00 (0.99, 1.00) | <0.01 |  | 1.01 (1.01, 1.02) | <0.01 |  | 0.99 (0.99,0.99) | <0.01 |
| Gender | 0.77 (0.70, 0.85) | <0.01 |  | - | - |  | - | - |
| Smoking | 1.20 (1.10, 1.31) | <0.01 |  | 1.30 (0.72, 2.37) | 0.39 |  | 1.17 (1.07,1.28) | <0.01 |
| Marital status | 1.28 (1.05, 1.54) | 0.01 |  | 1.40 (0.96, 2.03) | 0.08 |  | 1.13 (0.90,1.41) | 0.29 |
| Working type | 1.13 (1.01, 1.27) | 0.03 |  | 1.19 (1.00, 1.42) | 0.06 |  | 0.98 (0.84,1.14) | 0.78 |
| Education level | 0.85 (0.78, 0.94) | <0.01 |  | 0.71 (0.60,0.83) | <0.01 |  | 0.92 (0.82,1.03) | 0.13 |
| Physical activity | 1.09 (1.01, 1.18) | 0.03 |  | 1.04 (0.90,1.21) | 0.58 |  | 1.11 (1.01,1.22) | 0.03 |
| TG | 1.17 (1.13, 1.22) | <0.01 |  | 1.26 (1.16,1.35) | <0.01 |  | 1.14 (1.09,1.18) | <0.01 |
| TC | 1.03 (1.00, 1.06) | 0.03 |  | 1.02 (0.98,1.06) | 0.40 |  | 1.03 (0.99,1.07) | 0.14 |
| FBG | 1.02 (1.00, 1.05) | 0.04 |  | 1.05 (1.01,1.09) | 0.01 |  | 1.00 (0.98,1.03) | 0.79 |
| SBP | 1.00 (1.00, 1.00) | 0.04 |  | 1.00 (1.00,1.00) | 0.99 |  | 1.00 (1.00,1.01) | 0.07 |
| CRP | 1.01 (1.01, 1.02) | <0.01 |  | 1.02 (1.00,1.03) | 0.02 |  | 1.01 (1.00,1.02) | 0.01 |
| Cr | 1.00 (1.00, 1.00) | 0.01 |  | 1.00 (1.00,1.01) | 0.14 |  | 1.00 (1.00,1.00) | 0.06 |

**Notes:**

**Abbreviations:** BMI, Body Mass Index; CI: Confidence Interval; Cr, Creatinine; CRP, C-reactive Protein; FBG, Fasting Blood Glucose; OR, Odds Ratio; SBP, Systolic Blood Pressure; SUA, Serum Uric Acid; TC, Total Cholesterol; TG, Triglyceride.
