# Supplementary figures and images for: Serum Uric Acid Is a Mediator of the Association Between Obesity and Incident Nonalcoholic Fatty Liver Disease: A Prospective Cohort Study
Source: Front Endocrinol (Lausanne). 2021 May 13;12:657856. doi: 10.3389/fendo.2021.657856 (PMC8158156; doi:10.3389/fendo.2021.657856)

Supplemental Figure 1. The changes of serum parameters and BMI during follow up period.

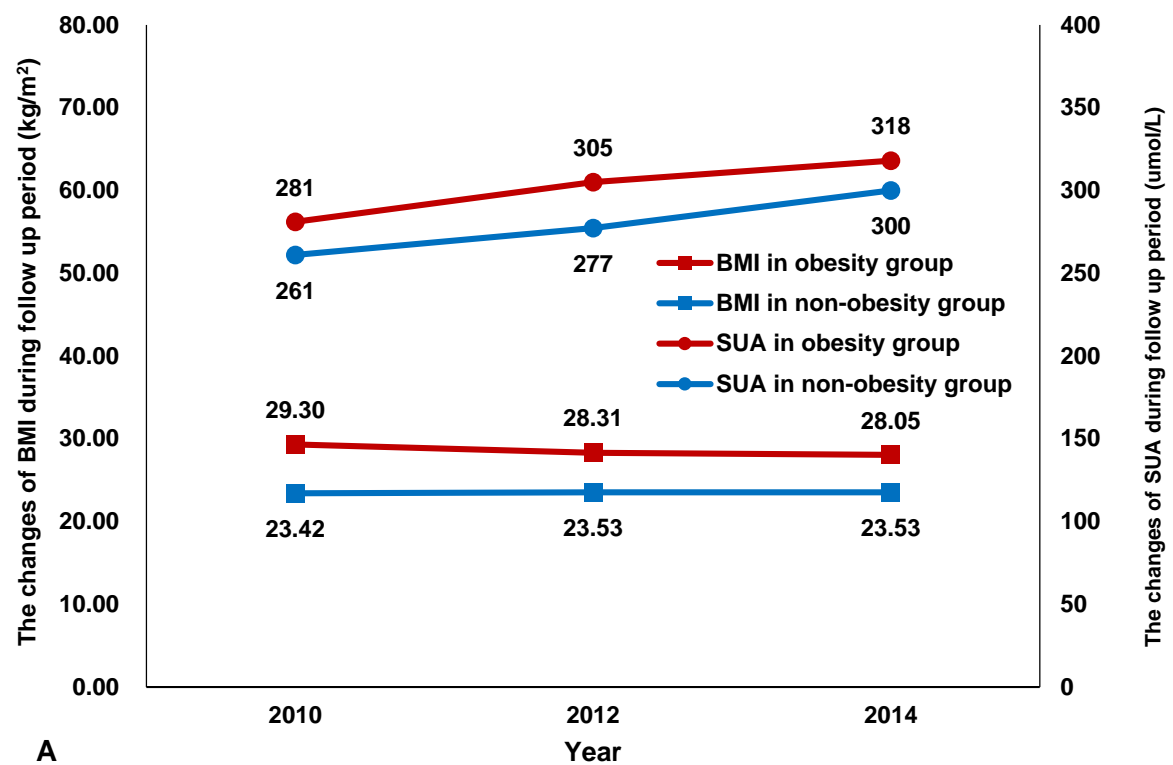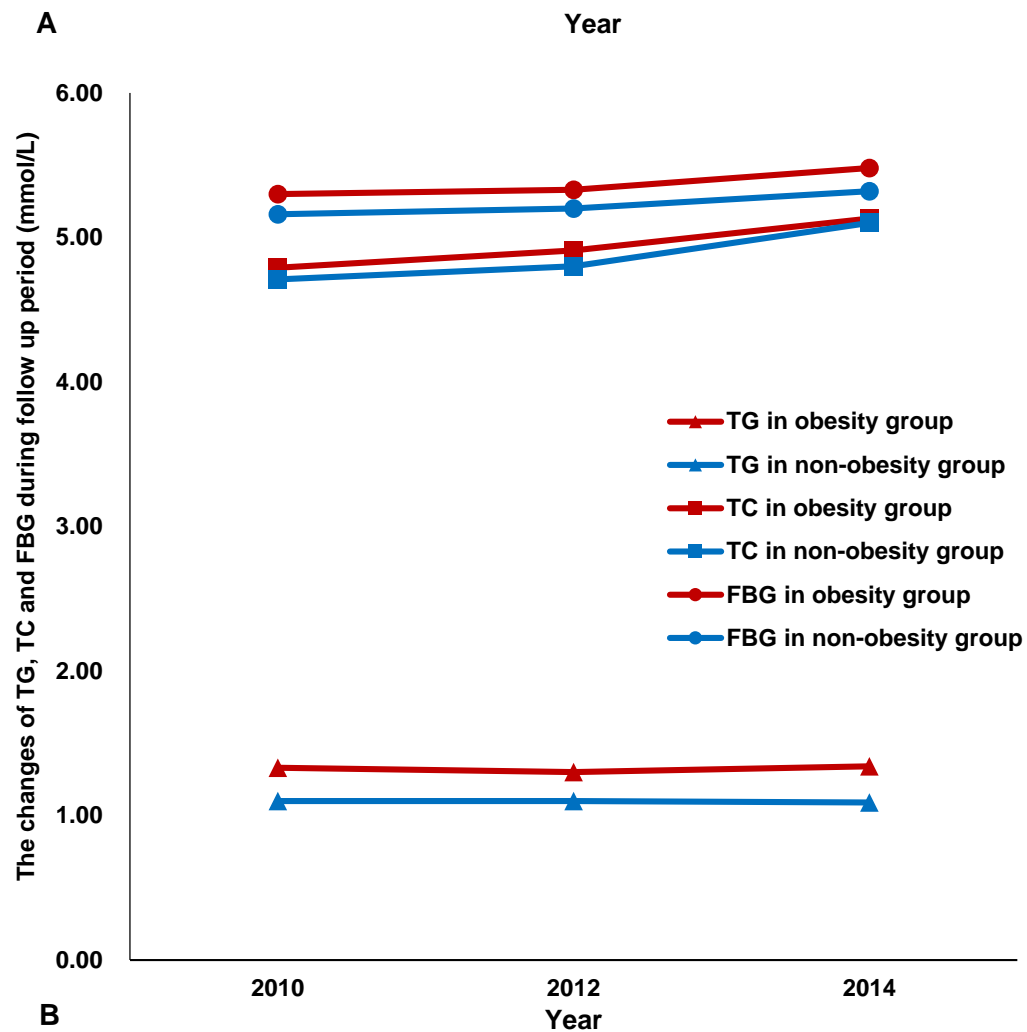

Supplement: Supplementary file 3 [file Image_1.pdf]
